# Eco-Efficient Fiber-Reinforced Concrete: From Mix Design to Fresh and Hardened State Behavior

**DOI:** 10.3390/ma18061245

**Published:** 2025-03-11

**Authors:** Ana Bergmann, Mohammed Nabil Eid, Mayra T. de Grazia, Sergio R. A. Dantas, Leandro F. M. Sanchez

**Affiliations:** 1 Department of Civil Engineering, University of Ottawa, Ottawa, ON K1N 6N5, Canada; meid014@uottawa.ca (M.N.E.); mtagl010@uottawa.ca (M.T.d.G.); leandro.sanchez@uottawa.ca (L.F.M.S.); 2 Department of Civil Engineering, École de Technologie Supérieure—ÉTS, Montreal, QC H3C 1K3, Canada; sergiorobe@me.com

**Keywords:** fiber-reinforced concrete (FRC), low-cement fiber-reinforced concrete, sustainability, fresh and hardened state

## Abstract

Fiber-reinforced concrete (FRC) mixtures often face challenges in the fresh state, which are typically addressed using high Portland cement (PC) content or chemical admixtures, obstructing sustainability efforts in the construction industry. Therefore, this study employs advanced mixed design techniques, specifically particle packing models (PPMs), to proportion eco-efficient FRC mixtures with reduced cement content (<300 kg/m^3^) while achieving desirable fresh and hardened state properties. Twelve low-cement (LC) FRC mixtures, containing limestone filler (LF) as an inert material and a partial replacement for PC, were designed with a water-to-cement ratio of 0.64, incorporating two fiber types (polypropylene and steel) at varying contents (0.5% and 1.0% by volume) and lengths (38 mm and 50 mm). PPM-designed mixtures used two coefficients of distribution (q-factors: 0.21 and 0.26) and were evaluated for fresh (VeBe time, slump, and rheology) and hardened (compressive strength and flexural performance) state properties. Results show that PPM-designed FRC mixtures achieved up to 70% higher compressive strength and up to 64% greater flexural capacity compared to conventional mixes (i.e., American Concrete Institute—ACI), despite using 20% less cement. Additionally, PPM mixtures exhibited higher VeBe times (up to 24 s) and yield stress, reflecting improved packing density, while demonstrating shear-thinning behavior for practical applications (i.e., pumped or vibrated concrete). Finally, the findings demonstrate that PPMs enable the development of eco-efficient, low-cement FRC mixtures with similar or improved hardened state performance and reduced environmental impact.

## 1. Introduction

Concrete remains the most widely used construction material globally, due to its mechanical properties, long-term durability, cost-effectiveness compared to other building materials (i.e., steel), and its ability to be cast into complex shapes. Despite these advantages, the production of concrete has a significant environmental footprint, contributing approximately 8% of global CO_2_ emissions, making it a significant contributor to greenhouse gas generation [1,2]. The environmental impact of concrete is further compounded by the surging global demand for PC, which has increased thirty times since 1950 and quadrupled since 1990 [2]. Given that each ton of PC produced releases nearly one ton of CO_2_ [3], there is an urgent need for measures to reduce PC content in concrete mixtures as a pathway toward sustainable construction practices.

Several approaches have been proposed to mitigate the environmental impact of cement-based materials. One widely adopted strategy involves the partial replacement of cement with supplementary cementitious materials (SCMs), such as metakaolin, fly ash and slag, which can enhance mechanical properties and durability while reducing CO_2_ emissions [4,5]. Another effective approach involves optimizing the particle size distribution (PSD) of powders and aggregates through advanced-mix proportioning methods, notably particle packing models (PPMs) [3,6]. While SCMs contribute chemically to the cementitious matrix, PPM-based designs optimize the physical arrangement of particles to achieve higher packing density, leading to reduced void spaces, enhanced material efficiency, and lower cement consumption [3,5].

Beyond PPMs, PC reduction in concrete may be achieved by using inert materials, so-called inert fillers [1,7]. These fillers are commonly used due to the physical benefits they bring to concrete, such as reduced porosity, commonly known as the “filler effect”. Furthermore, it has been found that limestone fillers (LFs) may present low reactivity with PC and thus change its hydration process due to the enhancement of nucleation sites [8]. The latter may result in an increase in the mechanical properties in the early ages and improve the overall rheological behavior of the material [8]. In fact, studies have demonstrated that PPM-based designs can achieve significant reductions in cement content when replaced by LF (i.e., up to 60%) while maintaining or even enhancing the mechanical properties and durability of concrete [3,9].

Other techniques, such as the incorporation of fibers, can play a crucial role in enhancing the mechanical properties of concrete by effectively bridging microcracks and distributing stresses, thereby achieving achieve the required strength and durability [10,11,12]. However, despite its numerous advantages in the hardened state, the addition of fibers can negatively impact fresh state behavior, leading to challenges in mixing, handling, and compaction that may compromise hardened properties [13,14]. Moreover, FRC mixes designed through conventional techniques (i.e., American Concrete Institute—ACI absolute volume method) [10] often require high paste contents to overcome flowability issues, which results in a significant increase in the amount of PC and thus raises the carbon footprint of the material [1,10,15,16].

In this context, this study aims to combine advanced mix design techniques (i.e., PPMs) with fiber incorporation to fully account for the complex rheological behavior of fresh FRC mixtures while exploring the potential of raw material properties. Therefore, this paper focuses on the development and analysis of eco-efficient fiber-reinforced concrete, by partially replacing cement with LF, covering aspects related to mix design, fresh state properties, and hardened performance. Finally, through a comprehensive investigation of the rheological properties of these mixtures, this work seeks to enhance the potential of PPMs and contribute to advancing sustainable concrete production.

## 2. Background

### 2.1. Particle Packing Models (PPMs)

PPMs are advanced mix-proportioning techniques; their main purpose is to decrease the number of intergranular voids in granular systems and thus increase packing density. The latter enables the development of concrete mixtures with a low amount of binder and enhanced behavioral in fresh and hardened states [3,17,18]. PPMs can be divided into two categories: discrete and continuous. Discrete PPMs consider a combination of multimodal distributions, aiming for the maximum packing of “n” discrete size classes of particles, so-called gap-graded systems [19]. Conversely, continuous PPMs intend to pack continuously sized particles available in the mixture with no gaps throughout the whole particle size distribution (PSD) [18,19]. The latter is considered compelling in concrete technology since most of the concrete components may be considered to present continuous PSDs. In 1907, Fuller and Thompson presented one of the first continuous PPMs, the well-known “Fuller-Thompson” ideal grading curve, according to Equation (1) [20]:

(1) $$CPFT{=(d/D)}^{n}\times 100$$

where CPFT is the cumulative percentage finer than d, d is the given particle size, D is the maximum particle size, and n is a distribution factor (i.e., from 0.45 to 0.5). Andreasen followed the works of Fuller and Thompson, and after some experimental testing and modeling, proposed an “ideal” curve known as the Andreasen model similar to Equation (1), assuming that the smallest particle is infinitesimally small [21]. Furthermore, Andreasen introduced the concept of a granulation image, emphasizing that the surrounding particles of different sizes should be similar, and suggested that the optimal “n” factor should range between 0.33 and 0.50, with a minimum particle size d0 equal to zero [22,23]. Funk and Dinger recognized that fine particles in real materials are finite in size and modified the initial Andreasen model considering the smallest particle size in the distribution. This approach gave rise to the so-called Alfred or modified Andreasen model, per Equation (2) [24]:

(2) $$CPFT=\left(\frac{d^{q}-d_{min}^{q}}{D_{max}^{q}-d_{min}^{q}}\right)\times 100$$

where CPFT is the cumulative percent finer than d, d is the given particle size, d_min_ is the minimum particle size, D_max_ is the maximum particle size, and q is the distribution factor. The q-factor may vary from 0.21 to 0.37, depending on the workability requirement. The higher the q-factor, the higher the amount of coarser particles in the system. In fact, after testing and modeling, the authors claimed that the densest particle packing could be achieved with a q-factor of 0.37, which would likely bring benefits to the short- and long-term hardened state properties of concrete along with a decrease in the binder content of the mixture. Otherwise, as the q-factor increases beyond 0.37, the porosity of the system rises instantly, yet the same trend is not observed for q values lower than 0.37 [19].

### 2.2. Fiber-Reinforced Concrete (FRC)

Fibers are usually added to concrete to counteract its brittle behavior, increasing its ductility and toughness, controlling crack propagation and delaying concrete failure [10,11,12]. Approximately 200,000 metric tons of fiber is currently used annually as reinforcement in concrete [25]. This is effective in partially or fully replacing conventional reinforcements in non-structural and structural elements, including industrial floors, pavements, beams, precast components, and tunnel linings [25,26]. Fibers of a wide range of materials and shapes are available. While steel fibers remain the most common, the use of polypropylene fibers has grown significantly in recent years [27].

The physical and mechanical characteristics of fibers, such as their length, diameter, and aspect ratio (length-to-diameter ratio), play a critical role in the performance of fiber-reinforced concrete (FRC) [28]. Higher aspect ratios typically enhance toughness and ductility by improving crack bridging and energy absorption. Volumetric diameter affects fiber dispersion, with larger diameters increasing interlocking but potentially reducing flowability. Specific gravity impacts mix density, while fiber shape (i.e., straight, hooked, or wavy) influences bond strength, pull-out resistance, and load transfer efficiency.

Therefore, despite its numerous advantages in the hardened state, the addition of fibers can negatively impact fresh-state behavior, leading to challenges in mixing, handling, and compaction that may compromise hardened properties [13,14]. FRC mixes designed through conventional techniques often require high paste contents to overcome flowability issues, which results in a significant increase in the amount of PC and thus raises the carbon footprint of the material [1,10,15,16]. Hence, optimizing FRC grading using PPMs along with inert fillers might be an appropriate approach to decreasing the environmental impact of FRC while achieving suitable fresh state behavior.

### 2.3. Binder Intensity Factor

Finally, quantifying the efficiency of concrete mixtures requires an evaluation of both performance and PC content. Damineli et al. (2010) introduced the binder intensity (bi) factor, an index designed to measure the eco-efficiency of concrete mixtures. The bi factor determines the amount of binder or PC required to achieve 1 MPa of a specified property, such as compressive strength. This metric provides a straightforward method for comparing the sustainability of different concrete formulations. The bi factor is calculated Equation (3) [29]:

(3) $$bi\;factor=\frac{BC}{f^{’}c}$$

where bi factor is binder intensity factor (kg/m^3^xMPa^−1^), BC is the amount of binder content (kg/m^3^) and f’c is the compressive strength (MPa) at a specific age (i.e., 28-day). bi factors commonly used in conventional concrete may vary from 9 to 14 kg/m^3^xMPa^−1^, regardless of their application [29]. Lower bi values indicate more eco-efficient mixtures, as they achieve the desired strength with reduced binder content.

## 3. Scope of the Work

This work aims to develop eco-friendly FRC mixtures designed through advanced techniques (i.e., continuous PPMs) with low cement content (<300 kg/m^3^) and suitable performance in both the fresh and hardened states. Unlike traditional FRC, which relies on high PC content, this approach optimizes particle packing for sustainability and strength. Therefore, twelve PPM mixtures were designed using the continuous Alfred model with varying coefficients of distribution (i.e., q-factors: 0.21 and 0.26), aiming to increase the proportion of finer particles, ensuring sufficient paste volume for fiber dispersion. Varying fiber parameters such as fiber types (i.e., steel and polypropylene), contents (i.e., 0.5%, and 1.0%), and lengths (i.e., 38 and 50 mm) were selected to evaluate their effects. The mixtures were assessed in the fresh (i.e., VeBe time, slump, and rheology) and hardened states (i.e., compressive and flexural strength). The performance of the PPM-proportioned mixtures was then compared with that of six control ACI conventionally designed mixes with the same fiber parameters. Finally, discussions on the suitability of using PPMs to proportion eco-efficient FRC regarding fresh and hardened properties were held.

## 4. Materials and Methods

### 4.1. Raw Material Characterization

Regarding the powders, GU PC cement, a fine gray powder, classified according to CSA A3001-18 [30], was selected as the binder. Its chemical composition, determined through X-ray fluorescence (XRF) analysis, is presented in Table 1. A limestone filler (LF), a light gray powder, presenting a similar PSD to that of cement was used to partially replace PC in the PPM mixtures.

The fine aggregate (FA) used in this study was natural sand, with a light brown to beige color, with a PSD conforming to ASTM C33 [31] and a fineness modulus of 2.49. The coarse aggregate (CA), predominantly gray, was crushed limestone sourced from Ottawa, ON, Canada, with a nominal maximum size (NMS) of 19 mm. The specific gravity of PC and LF was determined using the pycnometer test with helium gas (He). The specific gravity and absorption of FA and CA for moisture correction were determined according to ASTM C128-15 and ASTM C127-15 [32,33], respectively, and are summarized in Table 1.

**Figure 1:**
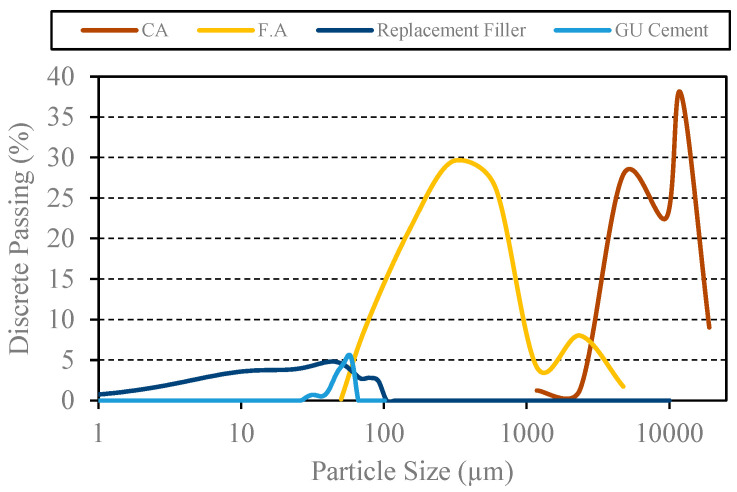
Particle size distribution of raw materials.

The PSDs of materials with particle sizes below 150 µm (i.e., PC and fillers) were measured using laser diffraction, while larger particles were characterized through mechanical sieving, following ASTM C33 [31]. For PPM-designed mixtures, the final size fractions were as follows: cement and fillers (≤150 µm), FA (150–300 µm, 300–600 µm, 600–1180 µm, 1180–2360 µm, and 2360–4750 µm), and CA (4750–9500 µm, 9500–12,500 µm, and 12,500–19,000 µm). For ACI-proportioned mixtures, the size fractions of the CA were kept consistent with those in the PPM mixtures. However, the FA was not fully controlled, as only the fineness modulus of the sand was considered. Figure 1 illustrates the PSD of all raw materials used in this study.

**Figure 2:**
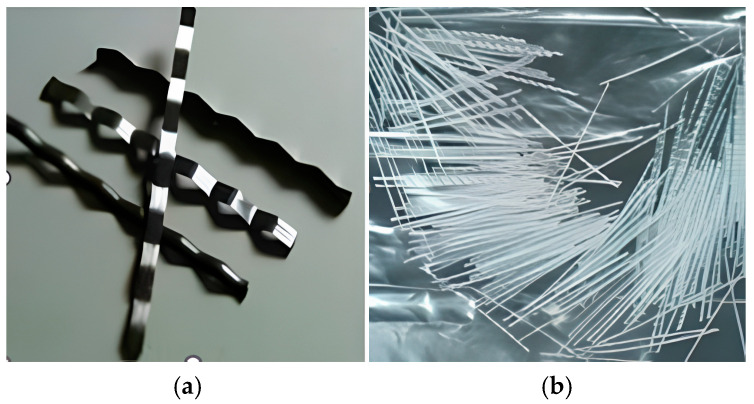
Fibers: (**a**) steel fibers (**b**) polypropylene fibers.

Table 2 summarizes the characteristics of the steel (S) and polypropylene (PP) fibers used in the mixtures, including length, diameter, aspect ratio, volumetric diameter, specific gravity, and fiber shape. Both fiber types have a wavy profile, with steel fibers being metallic silver and polypropylene fibers being white. The flexibility of PP fibers allows them to deform under stress, improving energy absorption and crack-bridging capacity, which enhances the material’s toughness. In contrast, the higher stiffness of steel fibers allows better load transfer across cracks, contributing to improved residual strength and post-cracking performance. Figure 2 visually illustrates the differences in fiber morphology.

### 4.2. Mix Design Method

Table 3 summarizes the mix proportions for all FRC mixtures, including cement, filler, fine aggregate (FA), coarse aggregate (CA), fibers, water, and admixtures. Two main sets of fiber-reinforced concrete (FRC) mixtures were prepared in this study: PPM-designed FRC mixtures and ACI-designed FRC mixtures. The designation of mixtures was based on the mix design method, fiber type, fiber volume fraction, fiber length, and q-factor. For example, “PPM-PP0.5-50-0.26” refers to a PPM-designed mixture containing 0.5% PP fibers with a length of 50 mm and a q-factor of 0.26.

#### 4.2.1. PPM-Designed FRC Mixtures

The first set consisted of twelve FRC mixtures designed using the PPM, with two q-factors (i.e., 0.21 and 0.26). The particle size range was set between 2 µm and 19 mm. The PC content was fixed at 300 kg/m^3^, with a water-to-cement ratio (w/c) of 0.64. A limestone filler, with a PSD similar to that of cement, was used to partially replace PC by volume.

Steel (S) and polypropylene (PP) fibers were incorporated at 0.5% and 1.0% volumetric fractions (Vf), and their inclusion in the PPM design followed the equivalent volume diameter (dv) approach. This method calculates the diameter of a fictitious sphere with the same volume as the fiber [34], as shown in Equation (5):

(4) dv=1.145 x Lfdf13 x df

where dv is the equivalent volume diameter (mm), Lf is the fiber length (mm), and df is the fiber diameter (mm). The calculated dv was used to include fibers in the granular skeleton of the mixture. The corresponding fiber volume fraction replaced an equivalent percentage of particles with diameters between 2360 µm and 4750 µm (i.e., fine aggregate fraction). To enhance the workability of PPM mixtures avoiding segregation, a polycarboxylate-based superplasticizer (SP) and a lignosulfonate-based plasticizer (P) were added. All PPM mixtures had the same admixture dosages (i.e., as a percentage of PC mass), which were determined to maintain a maximum VeBe time of 25 s, per [35,36,37].

#### 4.2.2. ACI-Designed FRC Mixture

The second set consisted of six control FRC mixtures proportioned using the ACI method [38], with adjustments per ACI 544.3R-08 [39]. These mixtures had a fixed PC content of 375 kg/m^3^ and a w/c of 0.64, consistent with the PPM mixtures. An SP admixture was also included, following the same dosage strategy used for the PPM mixtures.

### 4.3. Fresh and Hardened State Testing Methods

Fresh concrete samples were evaluated based on ASTM C172-17 [40], ensuring proper mixing, casting and curing procedures. The mixing process was conducted in a rotating drum mixer, starting with the dry materials (i.e., cement, limestone filler, fine aggregate, and coarse aggregate) followed by gradually adding water and chemical admixtures. Immediately after mixing, the fresh concrete properties were evaluated using standard workability and rheological tests.

**Figure 3:**
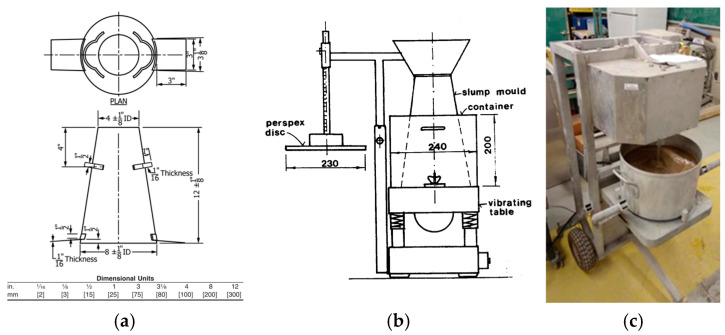
Fresh state property testing setups: (**a**) slump test [41], (**b**) VeBe test (adapted from [45]), and (**c**) IBB rheometer.

The slump test, performed per ASTM C143 [41] (Figure 3a), involved filling a standard cone in three layers, compacting each with 25 tamping strokes, and then lifting the cone to measure the slump height, indicating the mixture’s consistency and flowability. Additionally, the VeBe test, performed per EN 12350-3 [41,42] (Figure 3b), assessed the consistency of FRC by measuring the time required for a sample placed in a vibrating cylinder to fully settle and spread under a disk.

Rheological measurements were conducted through the IBB rotational rheometer (Figure 3c), proposed by Tattersall [43] and modified afterwards [44]. The rheological profile was determined based on a two-step process: (1) the speed of the impeller was increased in eight controlled stages up to a shear rate of approximately 43 rpm (i.e., acceleration process); (2) the rotation rate was decreased at the same stepwise rate until 2 rpm was reached, meaning that each stage had at least two complete center shaft revolutions. The main rheological parameters (i.e., viscosity and yield stress) were determined as a function of the shear stress. It is worth noting that yield stress is the minimum torque enabling a flow to the mixes, whereas viscosity (or apparent viscosity—AV) is considered in this work to be the ratio between torque and rotation at the first deceleration point of the shear stress–rate curve.

Next, cylindrical specimens (Ø100 mm × 200 mm) were prepared for compressive strength tests, while prismatic specimens (150 mm × 150 mm × 400 mm) were cast for flexural strength evaluation. To ensure proper compaction, the mixtures were placed in molds in two layers and consolidated using a vibrating table, with each layer compacted to remove air voids and achieve uniform density. Once cast, the molds were covered with plastic sheets to prevent excessive moisture loss and ensure adequate initial curing. After 24 h, the specimens were demolded, grounded and transferred to a moist curing chamber maintained at 23 ± 2 °C and at least 95% relative humidity.

**Figure 4:**
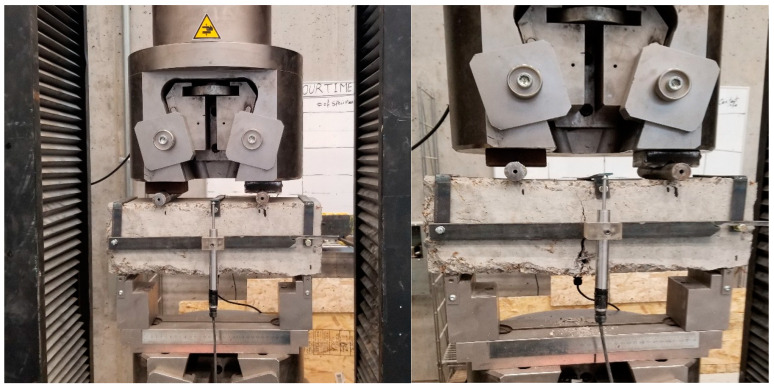
Flexural strength test conducted on UTM with a 1000 kN capacity by four-point loading at 28 days.

Regarding the hardened state, nine cylinders were tested for compressive strength tests at different ages (i.e., 7, 14, and 28 days), according to ASTM C39 [46], and two prisms were used for flexural analysis through the four-point loading test at 28 days, per ASTM C1609 [47] (Figure 4), for each mixture.

The load–deflection response was recorded during the flexural tests to evaluate key parameters such as peak load (PL), post-cracking behavior, and residual stress. Residual load and stress values were determined at net deflections of L/600 and L/150 to assess the effectiveness of fiber reinforcement. The modulus of rupture (MOR) was derived from the PL, while toughness was calculated as the area under the load–deflection curve up to 3 mm of deflection. Residual stress was further computed, considering the applied load (P), span length (L), and specimen dimensions at the respective deflection points. These parameters provide a comprehensive assessment of the mechanical performance of the FRC mixtures, particularly in terms of their ability to sustain stresses after cracking.

## 5. Results

### 5.1. Fresh State

Table 4 presents the results obtained for the slump test, VeBe test, and rheological characterization. The terms “applicable” and “non-applicable” indicate whether or not the rheology test could be performed on each mixture given the torque capacity of the IBB rheometer. The results will be explored in detail in this section.

**Figure 5:**
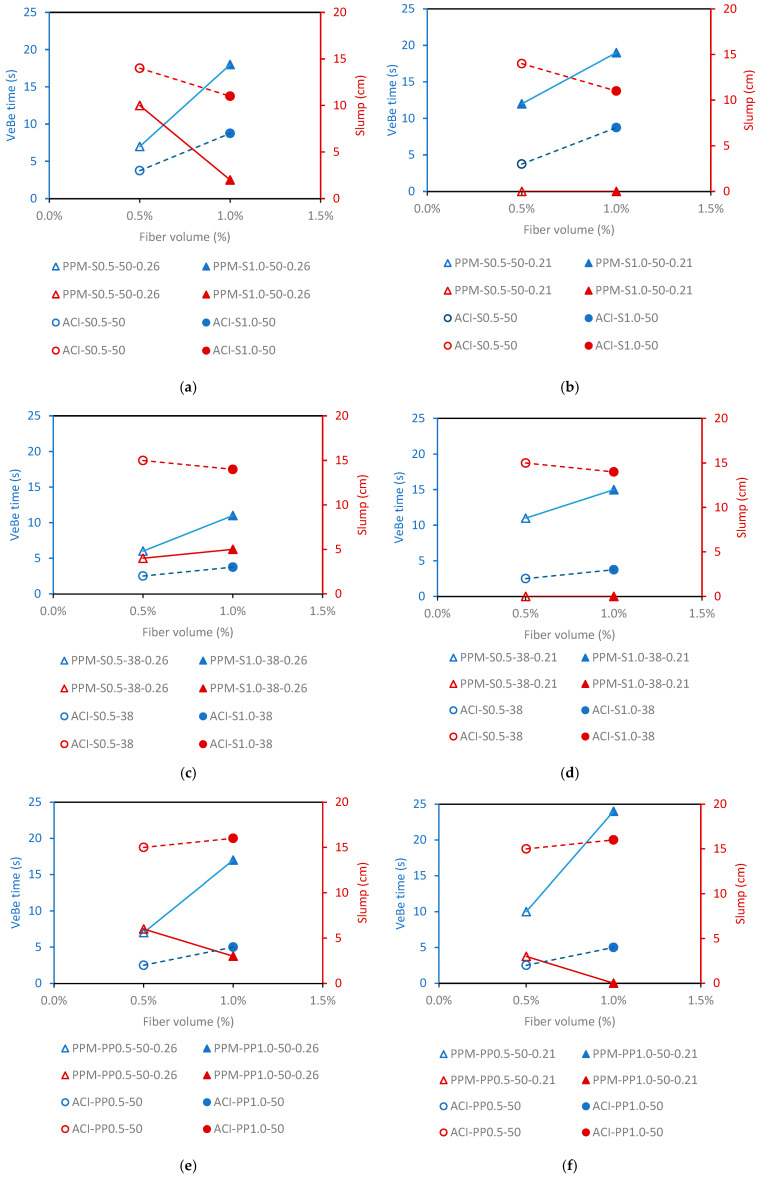
VeBe and slump tests of the distinct mixes as a function of fiber contents: (**a**) PPM (q-factor-0.26) and ACI mixtures—fibers: S-50 mm; (**b**) PPM (q-factor-0.21) and ACI mixtures—fibers: S-50 mm; (**c**) PPM (q-factor-0.26) and ACI mixtures—fibers: S-38 mm; (**d**) PPM (q-factor-0.21) and ACI mixtures—fibers: S-38 mm; (**e**) PPM (q-factor-0.26) and ACI mixtures—fibers: PP-50 mm; (**f**) PPM (q-factor-0.21) and ACI mixtures—fibers: PP-50 mm.

Figure 5 illustrates the relationship between VeBe time, slump, and fiber content for both PPM- and ACI-designed mixtures. Since VeBe time is an indicator of workability, shorter times reflect higher flowability, while slump measures the concrete’s resistance to deformation, with higher values indicating higher flow.

Overall, ACI mixtures exhibited shorter VeBe times compared to PPM mixtures. Increasing fiber content resulted in longer VeBe times across all mixtures, with a more pronounced impact observed in PPM mixtures. VeBe times for mixtures containing steel (S) fibers were generally similar to or slightly longer than those with polypropylene (PP) fibers, for the same fiber length (50 mm). Longer fibers consistently led to higher VeBe times in all mixtures, while shorter fibers (38 mm) reduced VeBe times, particularly at higher fiber contents. PPM mixtures designed with a distribution factor (q-factor) of 0.21, corresponding to a higher fine content, showed VeBe times that were either similar to or longer than those with a q-factor of 0.26.

In ACI mixtures incorporating 50 mm fibers, VeBe times ranged from 3 s for ACI-S0.5-50 to 7 s for ACI-S1.0-50. Conversely, mixtures with PP fibers had shorter VeBe times, ranging from 2 s for ACI-PP0.5-50 to 4 s for ACI-PP1.0-50. ACI mixtures with shorter fibers (38 mm) demonstrated the shortest VeBe times, at 2 s for ACI-S0.5-38 and 3 s for ACI-S1.0-38.

For PPM mixtures with a q-factor of 0.26 and 50 mm fibers, the VeBe times were 7 s for PPM-S0.5-50-0.26 and 20 s for PPM-S1.0-50-0.26. When using PP fibers, the VeBe times were 7 s for PPM-PP0.5-50-0.26 and 17 s for PPM-PP1.0-50-0.26. PPM mixtures with a q-factor of 0.21 exhibited higher VeBe times, ranging from 12 s for PPM-S0.5-50-0.21 to 20 s for PPM-S1.0-50-0.21, while PP fibers resulted in durations of 11 s for PPM-PP0.5-50-0.21 and 24 s for PPM-PP1.0-50-0.21. For mixtures incorporating 38 mm fibers, VeBe times were shorter, particularly at higher fiber contents. Durations ranged from 5 s for PPM-S0.5-38-0.26 to 12 s for PPM-S1.0-38-0.26, while for a q-factor of 0.21, the durations ranged from 12 s for PPM-S0.5-38-0.21 to 16 s for PPM-S1.0-38-0.21.

Regarding slump, ACI mixtures consistently displayed higher slump values, indicating lower consistency, compared to PPM mixtures. As fiber content increased, slump values decreased across all mixtures, with the effect being slightly more pronounced for S fibers and PPM mixtures. Fiber length also negatively impacted slump, particularly in PPM-designed mixtures. PPM mixtures with a q-factor of 0.21 showed lower slump values than those with a q-factor of 0.26, likely due to the higher fine content in the mix. Slump values ranged from 18 to 10 cm for ACI mixtures and from 10 to 0 cm for PPM-designed mixtures.

### 5.2. Rheological Characterization

Table 5 summarizes the rheological properties of ACI and PPM mixtures, presenting the minimum torque and apparent viscosity (AV) values for each mix. Minimum torque represents the yield stress, indicating the force required to initiate flow, while apparent viscosity reflects the ease of flow. It is worth noticing that the rheological properties of certain mixtures could not be measured due to the limitations of the rheometer (i.e., IBB) used in this study. The device was unable to accurately assess high-consistency (or high-viscosity) mixtures within its low-torque operating range. Consequently, rheological tests were not conducted for the following mixtures: ACI-S1.0-50, PPM-S1.0-50-0.26, PPM-S1.0-38-0.26, and all PPM mixtures with a q-factor of 0.21.

**Figure 6:**
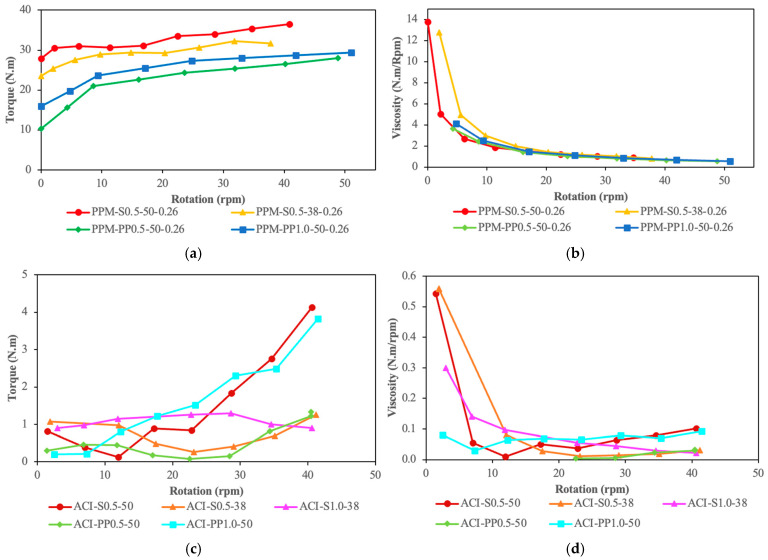
Rheological behavior of the mixtures. Torque–rotation curves for (**a**) PPM and (**c**) ACI mixtures; plastic viscosity–rotation curves for (**b**) PPM and (**d**) ACI mixtures.

Figure 6a,c show the relationship between torque and rotation for PPM and ACI mixtures, respectively, while Figure 6b,d depict the AV as a function of rotation for the same mixtures. ACI mixtures generally exhibited lower minimum torque values (i.e., yield stress) than PPM mixtures, though differences in AV were not as significant. Increasing fiber content led to higher minimum torque values across all mixtures, with the effect being more pronounced in PPM mixtures. Furthermore, mixtures containing S fibers consistently required higher minimum torque than those with PP fibers, regardless of fiber content or length. This difference was particularly noticeable in PPM mixtures. Fiber length also played a crucial role, with longer fibers leading to higher minimum torque in all cases.

For ACI mixtures with 50 mm fibers, the minimum torque recorded was 0.40 N·m for ACI-S0.5-50, while both ACI-PP0.5-50 and ACI-PP1.0-50 had registered torques of 0.20 N·m. Mixtures with shorter fibers (i.e., 38 mm) had minimum torque values of 0.26 N·m for ACI-S0.5-38 and 0.90 N·m for ACI-S1.0-38. In contrast, for PPM mixtures with a q-factor of 0.26 and 50 mm fibers, the minimum torque values were significantly higher, reaching 27.48 N·m for PPM-S0.5-50-0.26. For PP fibers, the values were comparatively lower, at 7.00 N·m for PPM-PP0.5-50-0.26 and 15.61 N·m for PPM-PP1.0-50-0.26. Shorter fibers (i.e., 38 mm) reduced the minimum torque, with that for PPM-S0.5-38-0.26 being 23.50 N·m.

When analyzing AV, ACI mixtures exhibited consistently lower values than PPM mixtures, though the differences were relatively small. For ACI mixtures, increasing fiber content led to higher AV values, particularly for those with PP fibers, whereas mixtures with short S fibers (i.e., 38 mm) displayed similar AV values regardless of fiber content. For PPM mixtures with PP fibers, AV values remained largely stable as fiber content increased.

In PPM mixtures, AV values were higher for those incorporating S fibers than for PP fibers, regardless of fiber content or length. Among the ACI mixtures, those with long S fibers showed the highest AV values, followed by PP fibers, while mixtures with short S fibers displayed the lowest AV. Fiber length negatively impacted AV in all mixtures, with this effect being more pronounced in ACI mixtures. For ACI mixtures with 50 mm fibers, AV values were recorded as 0.09 N·m/rpm for ACI-S0.5-50, and 0.03 and 0.08 N·m/rpm for ACI-PP0.5-50 and ACI-PP1.0-50, respectively. With shorter fibers (i.e., 38 mm), the AV values were 0.03 N·m/rpm for ACI-S0.5-38 and 0.02 N·m/rpm for ACI-S1.0-38.

For PPM mixtures with a q-factor of 0.26 and 50 mm fibers, the recorded AV values were 0.89 N·m/rpm for PPM-S0.5-50-0.26, while PPM-PP0.5-50-0.26 and PPM-PP1.0-50-0.26 had lower AV values of 0.52 and 0.50 N·m/rpm, respectively. For shorter fibers (i.e., 38 mm), PPM-S0.5-38-0.26 had a registered AV of 0.84 N·m/rpm.

Lastly, the viscosity of most ACI mixtures remained nearly constant as a function of applied torque, indicating relatively stable rheological behavior. In contrast, PPM mixtures displayed shear-thinning behavior, where viscosity decreased as the applied torque increased. This suggests that a specific amount of energy (i.e., yield stress or minimum torque) is required to initiate flow in PPM mixtures, reflecting their higher consistency and resistance to deformation.

### 5.3. Hardened State

Table 6 summarizes the hardened state results for both PPM and ACI mixtures, detailing compressive strength and flexural properties, which are analyzed and illustrated further in this section. Compressive strength (MPa) represents the maximum stress a material can withstand under compression. The coefficient of variation (CV, %) quantifies the variability in compressive strength results. The modulus of rupture (MOR, MPa) measures the material’s flexural strength, indicating its resistance to bending. Peak load (kN) corresponds to the maximum force sustained during flexural testing. Toughness (J) represents the total energy absorbed by the specimen until a specified deflection, reflecting its crack resistance and ability to deform under stress.

#### 5.3.1. Compressive Strength

**Figure 7:**
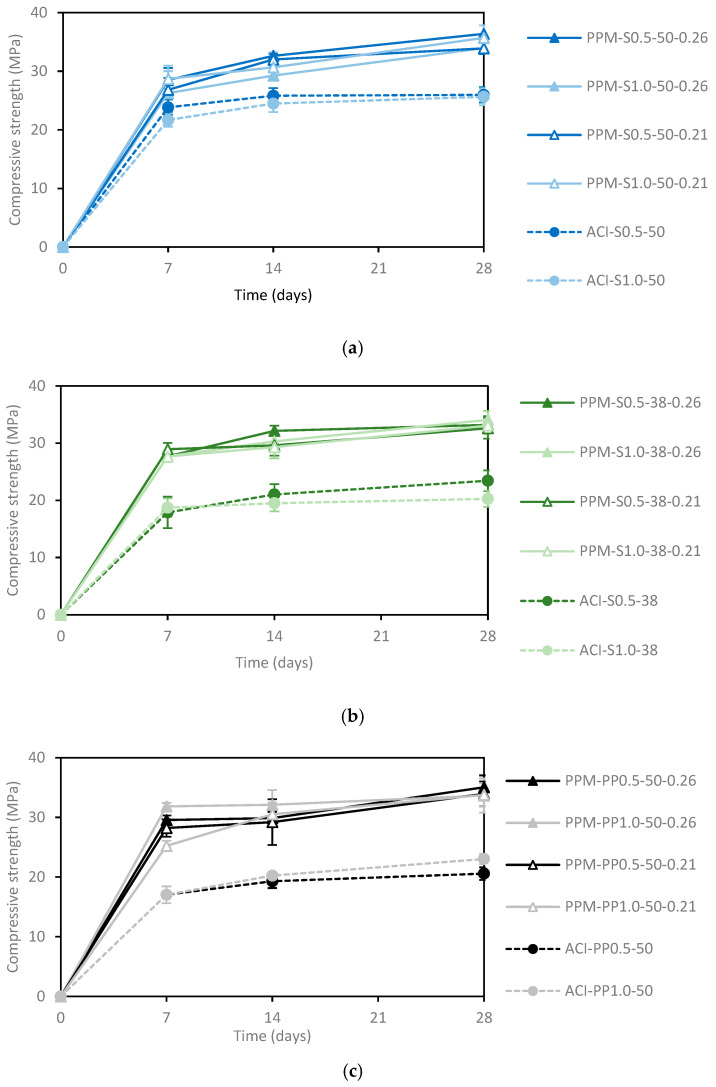
Compressive strength development as a function of time of concrete mixtures developed with (**a**) long S fibers, (**b**) short S fibers, and (**c**) PP fibers.

Figure 7a,c present the evolution of compressive strength over time, based on the average results of three specimens for the mixtures at 7, 14, and 28 days. The results indicate that PPM mixtures exhibited higher compressive strength compared to ACI mixtures. At 28 days, the compressive strength of PPM mixtures ranged from 32.6 to 36.4 MPa, while ACI mixtures achieved values between 20.3 and 26.0 MPa, representing an improvement of 30% to 70%, depending on the fiber type and length. It is important to highlight that both mix design methods maintained the same water-to-cement ratio, highlighting the better performance of PPM mixtures.

The increase in fiber content had a minimal impact on compressive strength for both PPM and ACI mixtures, with maximum variations of 13%. ACI mixtures containing long S fibers (i.e., 50 mm) showed an 18% improvement in compressive strength compared to those with short S fibers (i.e., 38 mm) or PP fibers. In contrast, this trend was not observed in PPM mixtures, where differences in compressive strength remained below 5%, regardless of fiber type or length.

Figure 7a shows that PPM mixtures with long S fibers (i.e., 50 mm) demonstrated an average compressive strength increase of 36% compared to ACI mixtures at 28 days. However, there was no significant difference in compressive strength between PPM mixtures with different q-factors (i.e., 0.26 and 0.21). Similarly, increasing the fiber content from 0.5% to 1.0% Vf had little effect, with variations of less than 7%.

Figure 7b reveals that PPM mixtures with shorter S fibers (i.e., 38 mm) achieved an average improvement of 53% over ACI mixtures at 28 days. For ACI mixtures, an increase in fiber content from 0.5% to 1.0% Vf led to a 15% rise in compressive strength. Conversely, variations in fiber content and q-factor did not significantly affect the compressive strength of PPM mixtures.

Figure 7c indicates that PPM mixtures with PP fibers displayed an average compressive strength increase of 57% compared to ACI mixtures. For ACI mixtures, an increase in PP fiber content resulted in a slight improvement of 12% in compressive strength. However, in PPM mixtures containing steel fibers, changes in the fiber content and q-factor had negligible influence, with variations in compressive strength results below 4%.

#### 5.3.2. Flexural Analysis

Table 7 shows the flexural strength (modulus of rupture, MOR) results for all mixtures. PPM mixtures with 0.5% Vf of long S fibers (i.e., 50 mm) exhibited MOR values that were 16% and 32% higher than those of ACI mixtures for q-factors of 0.26 and 0.21, respectively. However, at 1.0% Vf, PPM mixtures with long S fibers showed a 26% lower MOR than ACI mixtures with a q-factor of 0.26, while for a q-factor of 0.21, there was no significant difference. For mixtures with 0.5% Vf of short S fibers (i.e., 38 mm), the MOR differences between PPM and ACI mixtures were less than 10% for both q-factors. At 1.0% Vf, PPM mixtures with short S fibers showed an 18% lower MOR than ACI mixtures with a q-factor of 0.26, while the results were nearly identical for a q-factor of 0.21.

Conversely, PPM mixtures with PP fibers achieved higher MOR values than ACI mixtures across all conditions. For a q-factor of 0.26, MOR values were 33% and 36% higher than those of ACI mixtures with 0.5% and 1.0% Vf of PP fibers, respectively. For a q-factor of 0.21, the MORs increased by 55% and 64% over those of ACI mixtures with 0.5% and 1.0% Vf of PP fibers, respectively.

**Figure 8:**
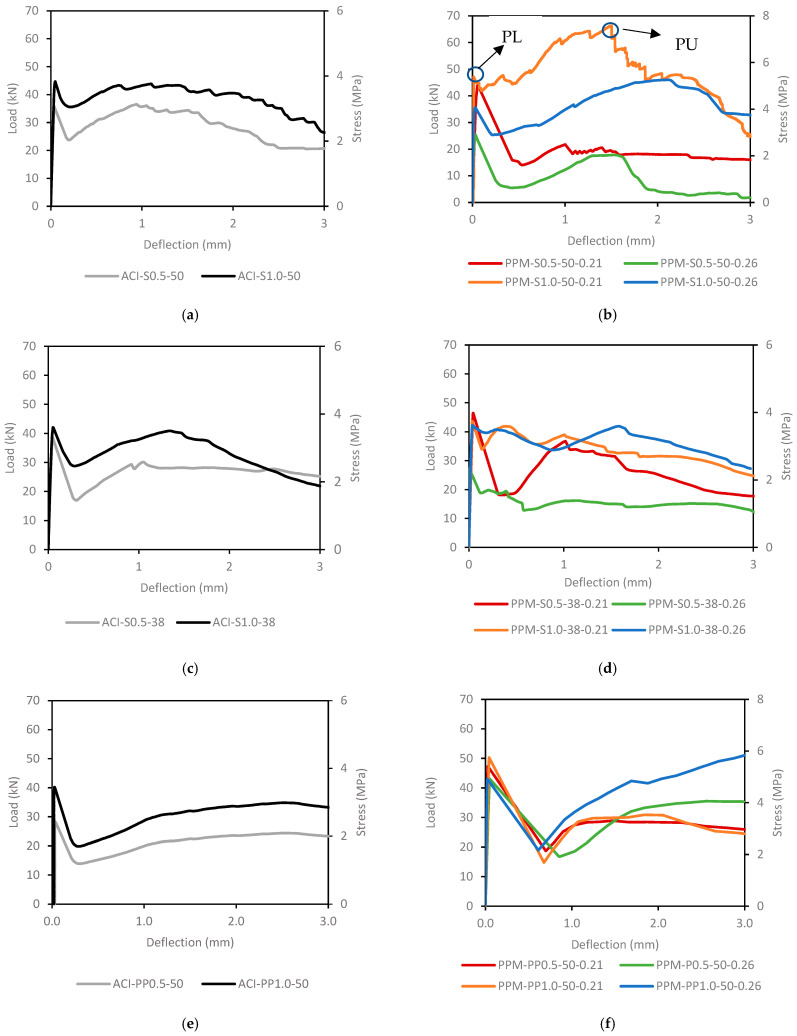
Flexural load–deflection curves of the ACI mixtures (**a**,**c**,**e**) and PPM mixtures (**b**,**d**,**f**). The region where the peak load (PL) is identified, representing the maximum load sustained before the first formation of a significant crack.

Figure 8 shows the flexural load–stress–deflection curves for all ACI and PPM mixtures. ACI mixtures demonstrated higher initial peak load (PL) and flexural stress at the first crack as fiber content increased from 0.5% to 1.0% Vf (Figure 8a). The highest PL and flexural stress were observed in ACI mixtures with 1.0% of long S fibers. After the first crack, ACI mixtures followed an ascending load–deflection curve, peaking before descending to failure. Most ACI mixtures with S fibers regained load capacity after cracking, except for the mixture with 0.5% of short S fibers (Figure 8c). In contrast, ACI mixtures with PP fibers did not regain load after cracking, regardless of fiber content (Figure 8e).

PPM mixtures with long S fibers (i.e., 50 mm) exhibited higher PL and flexural stress than ACI mixtures with a q-factor of 0.21, while these values were lower for a q-factor of 0.26 (Figure 8b). However, PPM mixtures with long S fibers showed improved load recovery in the post-cracking stage compared to ACI mixtures. For PPM mixtures with short S fibers (i.e., 38 mm), PL increased compared to ACI mixtures with a q-factor of 0.21, but no clear trend was observed for mixtures with a q-factor of 0.26 (Figure 8d). Additionally, PL improved with increasing fiber content only for mixtures with a q-factor of 0.26.

PPM mixtures with PP fibers consistently displayed higher PL values than ACI mixtures, regardless of fiber content or q-factor (Figure 8f). Increasing fiber content did not significantly affect PL in these mixtures. However, PPM mixtures with a q-factor of 0.21 showed PL values approximately 21% higher than those with a q-factor of 0.26. After the first crack, most PPM mixtures with PP fibers did not regain load capacity at the PL level, except for the mixture with 1.0% PP fibers and a q-factor of 0.26.

**Figure 9:**
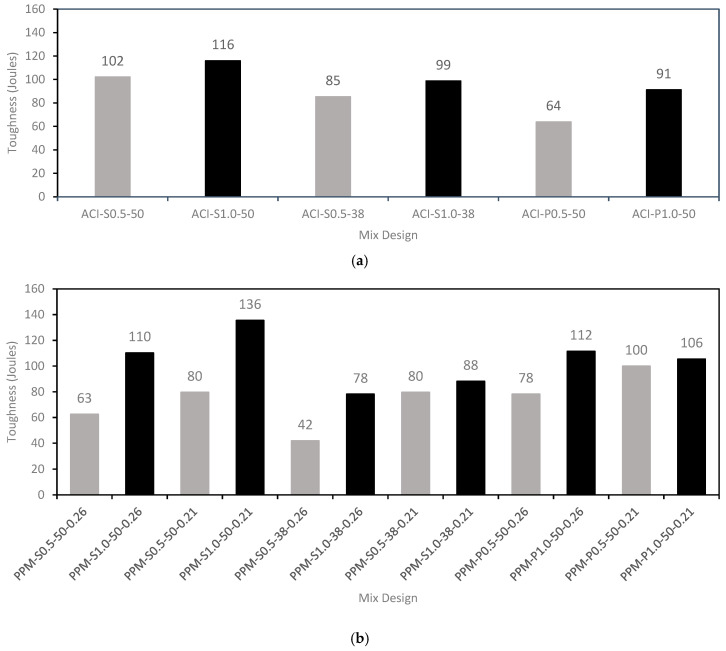
Toughness results of FRC mixtures: (**a**) ACI mixtures and (**b**) PPM mixtures. Results in gray represent mixtures developed with 0.5% of fibers, while results in black refers to mixtures developed with 1.0% of fibers.

Figure 9a,b show the toughness results (i.e., the material’s ability to absorb energy and deform without fracturing), which is calculated as the area under the load–deflection curve up to 3 mm of deflection, for ACI and PPM mixtures, respectively. PPM mixtures with 0.5% long S fibers exhibited lower toughness than ACI mixtures, with decreases of 38% and 22% for q-factors of 0.26 and 0.21, respectively. Conversely, PPM mixtures with 1.0% long S fibers and a q-factor of 0.21 showed the best performance among all mixtures.

PPM mixtures with short S fibers consistently displayed lower toughness than ACI mixtures, particularly for a q-factor of 0.26. In contrast, all PPM mixtures with PP fibers demonstrated higher toughness than ACI mixtures. Specifically, PPM mixtures with PP fibers and a q-factor of 0.26 achieved 22% higher toughness than ACI mixtures, regardless of fiber content. When using a q-factor of 0.21, toughness increased by 15% compared to that of ACI mixtures.

Table 8 displays the residual load and stress values of the distinct mixes at a net deflection of L/600, and L/150, reflecting the FRC’s ability to sustain stresses after cracking. These values are directly linked to the effectiveness of fiber reinforcement. Therefore, residual stress was calculated following Equation (5), per ASTM C1609 for the flexural performance of FRC [47]:

(5) $$f=\frac{PL}{bd^{2}}$$

where f is the strength (MPa), P is the load (kN), L is the span length in mm, b is the average width of the specimen at the fracture, and d is the average depth of the specimen at the fracture.

The results indicate that residual stress and load generally decreased as deflection increased for most mixtures with steel fibers, regardless of the mix design method. An exception was observed for PPM-S1.0-50-0.26, where residual stress increased from 3.43 MPa to 4.13 MPa as deflection increased from L/600 to L/150. Conversely, mixtures containing PP fibers showed an increase in residual stress and load at higher deflection values, regardless of mix-design method or fiber content.

For most PPM mixtures, residual stress increased with higher fiber content, except for mixtures with PP fibers and a q-factor of 0.21. In these cases, residual stress decreased from 2.36 MPa to 1.71 MPa and from 4.45 MPa to 3.00 MPa at deflections of L/600 and L/150, respectively. Similarly, ACI mixtures exhibited an increase in residual stress with higher fiber content, except for mixtures with short steel fibers (i.e., 38 mm).

Residual load results also showed an increase with higher fiber content for PPM mixtures with steel fibers. However, this trend was not consistent for mixtures with PP fibers or steel fibers designed with a q-factor of 0.26, where the residual load remained constant or decreased at L/600. ACI mixtures displayed a similar residual load pattern to PPM mixtures, except for those containing short steel fibers, where deviations were observed.

## 6. Discussion

### 6.1. Fresh State Performance

The addition of fibers often negatively affects the fresh state behavior of concrete mixtures due to increased friction and interlock effects between the fibers and coarse aggregate particles [13,14]. This interaction raises the mix consistency and reduces flowability, particularly at rest or under low torque conditions. However, in practical applications, conventional FRC is compacted using vibration (excluding self-leveling systems), which can significantly alter its flowability. Therefore, it is essential to perform tests, such as the VeBe test, that capture the ease of consolidation of FRC mixtures under vibration.

The analyzed results indicate that PPM-designed mixtures exhibited longer VeBe times with increased fiber content, a trend more pronounced for longer fibers. This can be attributed to the greater interlock effects between fibers and coarse aggregate particles. This behavior was expected, as ACI mixtures contained a much higher binder (PC) content, acting as a lubricant and facilitating flow under torque. In contrast, PPM mixtures had lower initial porosity (i.e., inversely proportional to PC content), resulting in reduced particle distances (i.e., powders, aggregates, and fibers), which hindered flow. Although S fibers, due to their higher density, might be expected to amplify this effect, the PP fibers used had a greater aspect ratio, behaving similarly in this case.

When comparing PPM mixtures with q-factors of 0.26 and 0.21, two distinct behaviors were observed. First, mixtures with S fibers and a q-factor of 0.21 exhibited a lower increase in VeBe time with higher fiber content. The last contains a higher fraction of fines (i.e., fillers) and fewer coarse aggregates, reducing friction between fibers and coarse aggregates. This suggests that using PPMs with lower q-factors may be advantageous for designing SFRC by decreasing the coarse aggregate fraction and minimizing fiber–aggregate interaction. Second, PP fiber mixtures with a q-factor of 0.26 showed a smaller reduction in flowability (as indicated by VeBe time) with increased fiber content compared to mixtures with a q-factor of 0.21. PP fibers, being more flexible, could better accommodate the spaces between coarse aggregates, primarily influencing paste flowability, unlike stiffer S fibers, which predominantly interact with coarse aggregates and aligned with previous findings [14,37].

Additionally, all PPM mixtures with a q-factor of 0.21 displayed longer VeBe times than their 0.26 counterparts, regardless of fiber type, content, or length. This can be attributed to the higher fines content in 0.21 mixtures, which reduced the distance between particles and increased the minimum torque required to initiate flow.

Although PPM-designed mixtures showed higher minimum torques (i.e., yield stress) than ACI, requiring greater effort to initiate flow, differences in apparent viscosity (AV) were not as significant despite their differing PC contents. PPM-designed FRC mixtures exhibited shear-thinning behavior, with viscosity decreasing as torque increased. This suggests that PPMs are a promising technique for proportioning vibrated or pumped FRC, as low viscosity under high-torque regimes is desirable for field applications.

### 6.2. Fresh State Modeling

Many models are available to describe the rheological behavior of cementitious systems. The most widely used model is the Bingham model, which assumes a linear relationship between shear stress and shear rate. However, not all concrete mixtures follow this linear behavior, as their viscosity may change depending on the applied torque [6,48]. For such cases, the Herschel–Bulkley (HB) model, shown in Equation (6), is more suitable. This is the most common non-linear model and provides a more accurate representation of the relationship between shear stress and yield stress in these systems [49].

(6) $$\tau =\tau ο+K\gamma^{n}$$

where τ is the measured torque (Pa.s), τo is the yield stress (Pa), describing the minimum yield stress required to initiate flow, *γ* is the speed of rotation (rev/m), K is the viscosity-constant parameter and n is the flow behavior factor; the latter are numerical parameters determined by the least square method.

Using the HB model, the rheological behavior of cementitious materials can be classified based on parameter n: shear thinning behavior (i.e., viscosity decreases with increasing torque) occurs when n < 1; shear thickening behavior (i.e., viscosity increases with increasing torque) occurs when n > 1; and for n = 1, the material exhibits Bingham behavior [3].

**Figure 10:**
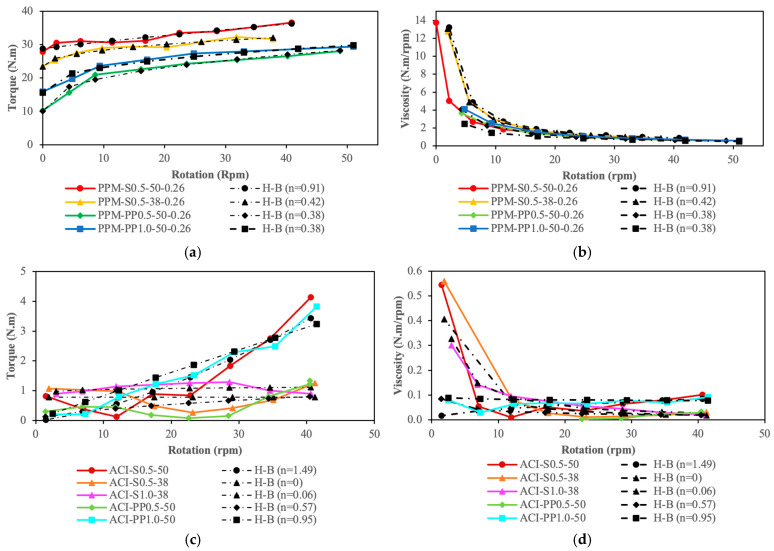
Modeled rheological behavior of the mixtures. Torque–rotation curves for (**a**) PPM and (**c**) ACI mixtures; plastic viscosity–rotation curves for (**b**) PPM and (**d**) ACI mixtures.

Therefore, Figure 10 displays the calculated HB rheological parameters and experimental results (i.e., minimum torque, and apparent viscosity). The results indicate that PPM mixtures exhibited lower relative error values compared to ACI mixtures, demonstrating the HB model’s competence in describing the fresh state behavior of PPM-mix-proportioned FRC. This suggests that the HB model can be effectively applied to design FRC mixtures with targeted yield stress and plastic viscosity ranges, optimized for specific applications.

### 6.3. Hardened State Performance

It is well established that the compressive strength of conventional concrete is primarily governed by the water-to-cement ratio, per Abram’s law, which determines the porosity of the hydrated system. However, John et al. [50] observed that Abram’s law does not apply to concrete mixtures with moderate-to-high amounts of inert fillers. In fact, the current study showed that although w/c ratios across both mix designs (i.e., ACI and PPMs) were kept the same, PPM mixtures achieved, on average, 47% higher compressive strength than ACI mixtures. These results suggest that although Abrams law is still the most critical factor controlling the compressive strength of conventional concrete mixtures, advanced techniques such as PPMs, which enhance the packing density of granular systems, can significantly improve mechanical performance, even with reduced cement content and increased filler use.

Fibers are primarily added to enhance crack control and ductility, rather than to improve compressive strength. However, confinement effects in FRC can sometimes enhance compressive strength, as noted by [51]. On the other hand, improper proportioning or compaction can increase porosity, reducing the compressive strength of FRC. Therefore, the observed variations in compressive strength in this study are likely influenced by a combination of these factors and the mix design method used (i.e., PPM or ACI). As consequence, FRC mixtures proportioned through PPMs also demonstrated comparable or superior flexural strength to ACI mixtures.

More specifically, when comparing the experimental results and empirical relations between the compressive strength and flexural strength (per Table 9) [52,53,54,55], deviations were observed, with most flexural strength results being underestimated (i.e., positive relative error) by the available empirical models, being more pronounced in the ACI mixtures.

**Figure 11:**
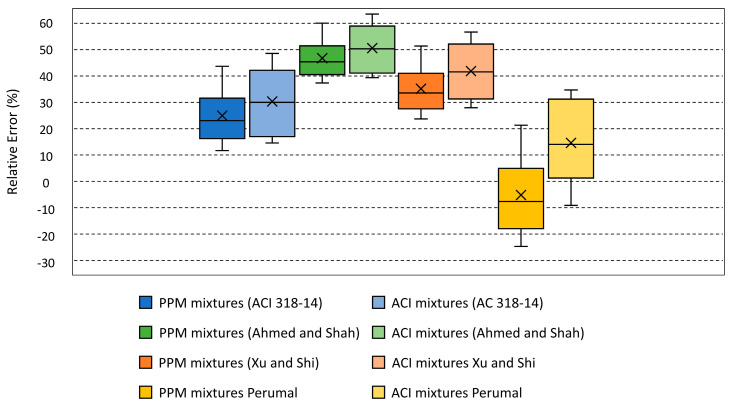
Relative error between the experimental flexural strength results and the empirical values.

In summary, Figure 11 displays the relative error between the obtained experimental results and available empirical relations.

For PPM mixtures, the underestimation may be attributed to the fact that the empirical models were developed for conventionally designed FRC. Therefore, the use of PPMs, along with the dilution effect of inert fillers, likely improved fiber dispersion within the mixtures, leading to higher experimental flexural strength. In contrast, the deviation in ACI mixtures may be related to the type of fibers used. While the empirical models were developed for straight and hooked-end fibers, the wavy-profile fibers may have contributed to the higher experimental flexural strength observed compared to the predicted values. Additionally, it can be noticed that the range of error is higher in the case of the ACI mixtures. This may be related to the fact that the developed empirical relations only account for the compressive strength of the mixtures; however, it was shown that flexural strength results obtained in this work generally increased as fiber content/and or length increased, whereas the compressive strength remained similar in most cases regardless of fiber content/and or length. Finally, it can be noticed that the experimental results of the PPM mixtures were overestimated by the empirical relation between compressive strength and flexural strength developed by [56], which may be attributed to the fact that the latter was developed for high-strength FRC (i.e., compressive strength > 45 MPa).

An enhancement was noticed in the toughness as a function of the fiber content/and or length. This impact is attributed to the confinement effect of fibers at high contents, which in turn provides the FRC with an enhanced crack growth arrest mechanism. Furthermore, the PPM mixtures with PP fibers exhibited higher toughness values than mixtures with S fibers, which may be related to the higher aspect ratio of the PP fibers and resulted in an increase in the total amount of fibers in the mixture at a fixed fiber content, improving the crack growth arrest mechanism. In addition, higher toughness values were noticed for PPM mixtures with a higher amount of inert fillers (i.e., q-factor 0.21). This impact may be attributed to the effective uniform dispersion of fibers in the mixtures caused by the diluting effect of limestone fillers, which was also noted by [57]. Finally, it is worth mentioning that all PPM mixtures exhibited improved load–deflection behavior when compared to the ACI mixtures, where in some cases the PPM mixture exhibited pseudo-deflection hardening behavior. The pseudo-deflection hardening behavior of concrete is associated with the appearance of multiple cracks increasing in density until composite ultimate flexural load is reached, changing the failure mode from quasi-brittle to ductile [58]. The latter may be attributed to the mix design approach adopted (i.e., PPMs) characterized by a theoretically increased packing density, accompanied by the dilution effect of inert fillers which improving the fiber dispersion efficiency and ultimately the concrete load–deflection behavior. This performance was similar to that of the load–deflection behavior of high-performance FRC noted by [51]. Furthermore, FRC pseudo-strain hardening behavior was also noticed in some PPM mixtures where the residual stress increased as the deflection rose. Finally, some mixtures displayed similar residual load values regardless of the fiber content; this could be related to the reinforcement efficiency at low fiber contents when subjected to low deflection values.

### 6.4. Quantification of Efficiency Indexes in Concrete

#### 6.4.1. Mobility Parameters

In order to better evaluate concrete behavior, two mobility parameters were used to account for the particle distance effects: the interparticle separation distance (IPS) and maximum paste thickness (MPT) [3,9]. These parameters are often used in combination with PPMs to help explain the behavior of highly packed systems such as concrete in the fresh and hardened states.

IPS is the average distance separating fine particles in the cement paste, and it represents the minimum amount of fluid (i.e., water) among particles [59,60]. Thus, it is assumed that there is no particle agglomeration, and a part of the fluid in the paste fills the voids and covers the surface of particles, whereas the remaining fluid builds up the separating layer of particles [60]. IPS is described in Equation (7):

(7) $$IPS=\frac{2}{VSA}\left[\frac{1}{Vs}-\frac{1}{\left(1-P_{of}\right)}\right]$$

where IPS is the interparticle separation distance, VSA is the volumetric surface area of the powder (m^2^/cm^3^), Vs is the volumetric solid fraction of the powder, and P_of_ is the powder pore fraction under maximum packing conditions. It is worth mentioning that in this work, powder is described a materials with a particle size smaller than 150 μm.

The maximum paste thickness (MPT) provides the maximum separating distance of particles among the aggregates. It represents the amount of cement paste between two adjacent particles and is described by Equation (8) [59,61]. MPT can be applied to better understand the flowability properties of concrete mixtures [62].

(8) $$MPT=\frac{2}{{VSA}_{c}}\left[\frac{1}{V_{sc}}-\frac{1}{\left(1-P_{ofc}\right)}\right]$$

where MPT is the maximum paste thickness, VSA_c_ is the volumetric surface area of the aggregate fraction (m^2^/cm^3^), V_sc_ is the volumetric solid fraction of the aggregate fraction, and P_ofc_ is the aggregate pore fraction under maximum packing conditions.

**Figure 12:**
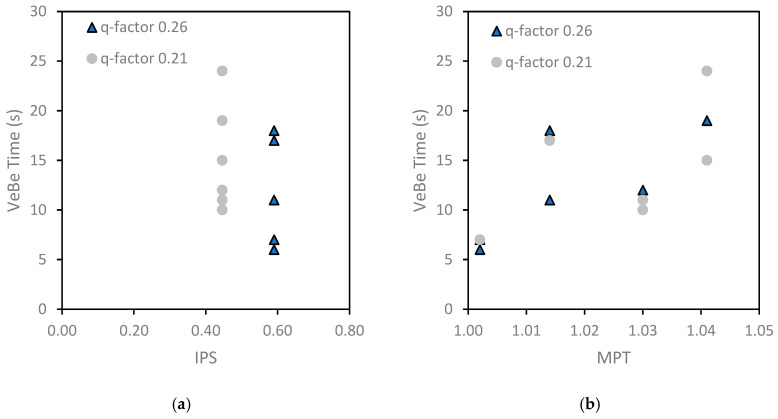
Relationship between (**a**) IPS and VeBe time (s), and (**b**) MPT and VeBe time (s).

Therefore, Figure 12a,b illustrate the relationship between the mobility parameters (i.e., IPS and MPT) and VeBe time for different q-factors in the PPM mixtures, with the aim to enhance understanding of the fresh state. Mixtures with the same q-factor had identical IPS values, but VeBe time decreased as IPS increased when the q-factor rose from 0.21 to 0.26, indicating improved flowability due to increased particle separation. For MPT, VeBe time increased with higher MPT values, as larger paste thickness reduced aggregate friction. However, no consistent relationship between q-factor and MPT was observed.

**Figure 13:**
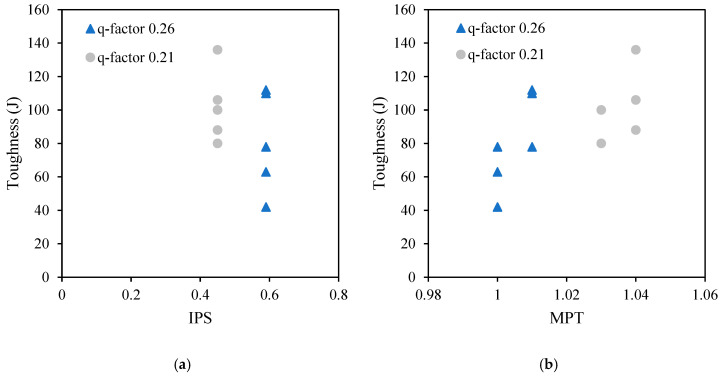
Relationship between (**a**) IPS and toughness (J), and (**b**) MPT and toughness (J).

Next, toughness was analyzed in relation to the mobility parameters for different q-factors, as shown in Figure 13. No consistent trend was observed between toughness and IPS or MPT; however, mixtures with higher q-factors (i.e., 0.26) generally exhibited lower toughness’ compared to those with lower q-factors (i.e., 0.21). This behavior suggests that despite the toughness of IPS and MPT, the q-factor plays a more prominent role in optimizing the mix. Additionally, the fiber characteristics, including type and length, likely contribute to their variability, as longer fibers and PP type may enhance energy absorption but also introduce more variability in the results.

#### 6.4.2. Efficiency Factor of FRC

Therefore, to account for the effect of fibers in FRC, a new parameter, the fiber matrix factor (FMF), is proposed by the authors. FMF describes FRC properties by combining the IPS, MPT, and fiber characteristics, as expressed in Equation (9):

(9) $$FMF=\frac{IPS\times MPT}{FF}$$

where FMF is the fiber matrix factor (dimensionless), IPS is the interparticle separation distance, MPT is the maximum paste thickness, and FF is the fiber factor.

The fiber factor (FF), introduced by [14], correlates fresh state properties of FRC with the hardened ones, and is defined by Equation (10):

(10) $$FF=V_{f}\times \left(\frac{L_{f}}{d_{f}}\right)$$

where FF is the fiber factor, V_f_ is the fiber content (%), L_f_ is the fiber length (mm), and d_f_ is the fiber diameter (mm).

**Figure 14:**
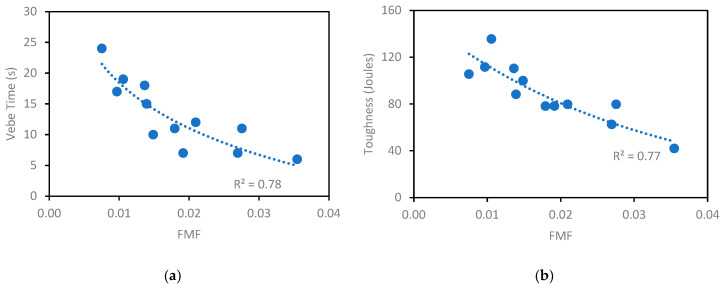
Relationship between FMF and (**a**) VeBe time (s) and (**b**) toughness (j).

Figure 14a,b explore the relationship between FMF and VeBe time, and between FMF and toughness. The PPM mixtures displayed a drop in VeBe time with the rise in FMF and the same trend was observed for toughness, which decreased with the increase in FMF. This behavior suggests that higher FMF values reduce packing efficiency, negatively affecting toughness while enhancing flowability. The R^2^ values of 0.78 and 0.77 for VeBe time and toughness, respectively, indicate a strong correlation, particularly given the multifactorial nature of FRC mixtures, which involve fiber content, type, aspect ratio, q-factors, interparticle separation, and paste thickness. While some scatter is expected due to these interactions, the observed correlations suggest that FMF effectively captures key trends in fresh and hardened state behavior. These findings indicate that FMF is a promising parameter for describing the fresh and hardened state behavior of FRC mixtures designed using PPMs.

Table 10 presents a summary of the efficiency index values for PPM mixtures, along with their corresponding VeBe times, toughness, and compressive strengths.

### 6.5. Eco-Efficiency of FRC Mixtures

**Figure 15:**
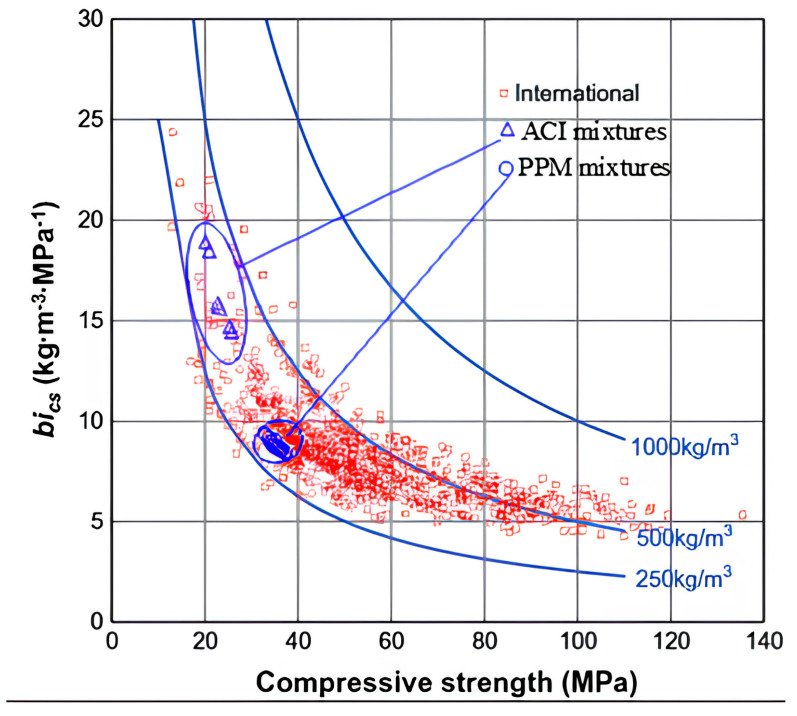
Relationship between binder intensity and compressive strength at 28 days; taken from international records adapted from [29].

Figure 15 presents a summary of international records correlating bi factors with compressive strength. The bi factor represents the amount of binder content required to achieve a compressive strength of 1 MPa, while compressive strength (f’c) quantifies the material’s ability to withstand axial loads, as previously defined in Section 2.3. The bi results obtained in this work through both PPM and ACI methods are also included in the plot for comparison purposes. Analyzing the plot below, one can see that most of the concrete produced for critical infrastructure worldwide (i.e., 20–40 MPa) is proportioned with moderate-to-high amounts of PC and thus yields high bi factors (i.e., 10 kg.m^3^.MPa^−1^) [29]. The latter is even more critical for FRC mixtures designed with conventional procedures such as ACI, where extremely high bi values of around 14.5 to 18.5 kg.m^3^.MPa^−1^ may be achieved. However, the use of advanced techniques such as continuous PPM may efficiently lower the bi values of FRC mixtures while maintaining or enhancing performance in the fresh and hardened states; hence, PPMs seem to be a suitable way to proportion eco-efficient FRC.

## 7. Conclusions

This study successfully developed low-carbon-footprint FRC using PPM mix proportioning methodology, achieving a balance of desirable properties in both fresh and hardened states. As a result, the following conclusions were drawn:

- PPM-designed FRC achieved up to 70% higher compressive strength and up to 64% superior flexural properties compared to ACI-designed mixtures, despite maintaining the same water-to-cement ratio.
- PPM mixtures exhibited higher VeBe times and minimum torque values, reflecting increased resistance to flow due to enhanced packing density and interaction between fibers, aggregates, and fines. However, their shear-thinning behavior indicate their suitability for target ranges of yield stress and plastic viscosity (i.e., pumped or vibrated concrete).
- PP fibers improved toughness and residual stress compared to S fibers in PPM mixtures, attributed to their higher aspect ratio and better dispersion. Increased fiber content further enhanced crack resistance, particularly in mixtures with higher inert filler (LF) content (q-factor 0.21).
- An improved fiber matrix factor (FMF) was developed, integrating mobility parameters such as interparticle separation distance (IPS) and maximum paste thickness (MPT), to better predict and control the behavior of PPM-mix-proportioned FRC.
- PPM-designed mixtures achieved lower binder intensity (bi) factors than ACI mixtures, aligning with sustainability goals while maintaining mechanical performance.

For future work, further optimization of mix proportioning (i.e., refining q-factors for different fiber types and dosages), a broader material scope (i.e., including alternative non-inert fillers, such as SCMs), assessments of long-term durability, and validation through large-scale applications are needed to enhance the applicability of PPM-based FRC.

## Figures and Tables

**Table 1:** Raw material characteristics and chemical composition (according to the manufacturer).

Material	Compound Content (%)									Blaine (m^2^/kg)	Loss on Ignition (%)	Specific Gravity (g/cm^3^)	Absorption (%)
	Na_2_O	MgO	Al_2_O_3_	SiO_2_	SO_3_	K_2_O	CaO	CaCO_3_	MgCO_3_				
GU Cement	0.14	2.66	4.92	19.53	3.86	0.77	61.91	-	-	381.46	2.24	3.15	-
LF—Replacement Filler	-	-	-	-	-	-	-	-	-	-	-	2.66	-
FA	-	-	-	-	-	-	-	-	-	-	-	2.73	0.79
CA	-	-	-	-	-	-	-	-	-	-	-	2.73	0.54

**Table 2:** Characteristics of fibers (according to the manufacturer).

Type of Fiber	Length (mm)	Diameter (mm)	Aspect Ratio	Volumetric Diameter (mm)	Specific Gravity (g/cm^3^)	Fiber Shape
Steel (S)	50	1.14	44	4.6	7.85	Wavy profile
Steel (S)	38	1.14	33	4.2	7.85	
Polypropylene (PP)	50	0.81	62	3.67	0.91	

**Table 3:** Mix proportions of FRC.

Mixtures	Cement (kg/m^3^)	Filler (kg/m^3^)	FA (kg/m^3^)	CA (kg/m^3^)	Fibers (kg/m^3^)	Water (kg/m^3^)	Admixtures (SP + P)
PPM-S0.5-50-0.26	300	178	975	731	39	192	0.4% + 0.2%
PPM-S0.5-38-0.26					39		
PPM-PP0.5-50-0.26					4.5		
PPM-S0.5-50-0.21		277	961	645	39		
PPM-S0.5-38-0.21					39		
PPM-PP0.5-50-0.21					4.5		
PPM-S1.0-50-0.26		178	961	731	79		
PPM-S1.0-38-0.26					79		
PPM-PP1.0-50-0.26					9		
PPM-S1.0-50-0.21		277	947	645	79		
PPM-S1.0-38-0.21					79		
PPM-PP1.0-50-0.21					9		
ACI-S0.5-50	375	-	567	1115	38	240	0.2% + 0.0%
ACI-S0.5-38					38		
ACI-PP0.5-50					4.5		
ACI-S1.0-50			553		79		
ACI-S1.0-38					79		
ACI-PP1.0-50					9		

**Table 4:** Fresh state results.

Mixture	VeBe Test (s)	Slump Test (cm)	Rheological Characterization
PPM-S0.5-50-0.26	7	10	Applicable
PPM-S0.5-38-0.26	6	4	Applicable
PPM-PP0.5-50-0.26	7	6	Applicable
PPM-S0.5-50-0.21	12	0	Non-applicable
PPM-S0.5-38-0.21	11	0	Non-applicable
PPM-PP0.5-50-0.21	10	3	Non-applicable
PPM-S1.0-50-0.26	18	2	Non-applicable
PPM-S1.0-38-0.26	11	5	Non-applicable
PPM-PP1.0-50-0.26	17	3	Applicable
PPM-S1.0-50-0.21	19	0	Non-applicable
PPM-S1.0-38-0.21	15	0	Non-applicable
PPM-PP1.0-50-0.21	24	0	Non-applicable
ACI-S0.5-50	3	14	Applicable
ACI-S0.5-38	2	15	Applicable
ACI-PP0.5-50	2	15	Applicable
ACI-S1.0-50	7	11	Non-applicable
ACI-S1.0-38	3	14	Applicable
ACI-P1.0-50	4	16	Applicable

**Table 5:** Rheological properties of the analyzed mixtures.

Mixture Name	Measured Properties	
	Minimum Torque (N.m)	Apparent Viscosity (N.m/rpm)
PPM-S0.5-50-0.26	27.48	0.89
PPM-S0.5-38-0.26	23.50	0.84
PPM-0.5PP-50-0.26	7.00	0.52
PPM-1.0PP-50-0.26	15.61	0.51
ACI-S0.5-50	0.40	0.09
ACI-S0.5-38	0.26	0.03
ACI-S1.0-38	0.90	0.02
ACI-PP0.5-50	0.20	0.03
ACI-PP1.0-50	0.20	0.08

**Table 6:** Hardened (i.e., compressive strength and flexural properties) state results of PPM and ACI mixtures.

Mixture	Compressive Strength (MPa)	CV (%)	Modulus of Rupture—MOR (MPa)	Peak Load (kN)	Toughness (J)
PPM-S0.5-50-0.26	36.4	4	4.3	24.1	63
PPM-S1.0-50-0.26	33.9	3	4.5	35.1	110
PPM-S0.5-50-0.21	33.9	3	4.9	42.5	80
PPM-S1.0-50-0.21	35.7	1	6.5	47.4	136
PPM-S0.5-38-0.26	33.2	5	4.2	24.2	42
PPM-S1.0-38-0.26	34.1	4	4.1	41.2	78
PPM-S0.5-38-0.21	32.6	6	4.3	44.1	80
PPM-S1.0-38-0.21	33.1	3	5.2	43.5	88
PPM-PP0.5-50-0.26	35.0	6	4.4	41.2	78
PPM-PP1.0-50-0.26	33.7	8	5.3	41.3	112
PPM-PP0.5-50-0.21	33.9	6	5.1	48.1	100
PPM-PP1.0-50-0.21	33.8	6	6.4	51.9	106
ACI-S0.5-50	26.0	5	3.7	32.2	102
ACI-S1.0-50	25.6	6	6.1	44.6	116
ACI-S0.5-38	23.4	8	4.7	39.1	85
ACI-S1.0-38	20.3	7	5.0	41.1	99
ACI-PP0.5-50	20.6	5	3.3	28.20	64
ACI-PP1.0-50	23.0	4	3.9	40.26	91

**Table 7:** Hardened (i.e., compressive strength and flexural properties) state results of distinct mixtures.

Mixture	Compressive Strength (MPa)	CV (%)	Modulus of Rupture—MOR (MPa)	Peak Load (kN)	Toughness (J)
PPM-S0.5-50-0.26	36.4	4	4.3	24.1	63
PPM-S1.0-50-0.26	33.9	3	4.5	35.1	110
PPM-S0.5-50-0.21	33.9	3	4.9	42.5	80
PPM-S1.0-50-0.21	35.7	1	6.5	47.4	136
PPM-S0.5-38-0.26	33.2	5	4.2	24.2	42
PPM-S1.0-38-0.26	34.1	4	4.1	41.2	78
PPM-S0.5-38-0.21	32.6	6	4.3	44.1	80
PPM-S1.0-38-0.21	33.1	3	5.2	43.5	88
PPM-PP0.5-50-0.26	35.0	6	4.4	41.2	78
PPM-PP1.0-50-0.26	33.7	8	5.3	41.3	112
PPM-PP0.5-50-0.21	33.9	6	5.1	48.1	100
PPM-PP1.0-50-0.21	33.8	6	6.4	51.9	106
ACI-S0.5-50	26.0	5	3.7	32.2	102
ACI-S1.0-50	25.6	6	6.1	44.6	116
ACI-S0.5-38	23.4	8	4.7	39.1	85
ACI-S1.0-38	20.3	7	5.0	41.1	99
ACI-PP0.5-50	20.6	5	3.3	28.20	64
ACI-PP1.0-50	23.0	4	3.9	40.26	91

**Table 8:** Residual load and stress values of distinct mixes.

Mixture	P_600_^d^ kN	F_600_^d^ MPa	P_150_^d^ kN	F_150_^d^ MPa
PPM-S0.5-50-0.26	30.30	2.41	16.32	1.93
PPM-S1.0-50-0.26	29.00	3.43	34.88	4.13
PPM-S0.5-50-0.21	23.32	2.77	18.93	2.24
PPM-S1.0-50-0.21	48.26	5.72	33.50	3.97
PPM-S0.5-38-0.26	18.58	2.20	14.83	1.76
PPM-S1.0-38-0.26	24.60	2.92	22.94	2.72
PPM-S0.5-38-0.21	17.45	2.07	11.97	1.42
PPM-S1.0-38-0.21	32.20	3.81	23.56	2.79
PPM-PP0.5-50-0.26	16.58	1.97	32.26	3.82
PPM-PP1.0-50-0.26	17.66	2.10	41.31	4.90
PPM-PP0.5-50-0.21	19.92	2.36	37.50	4.45
PPM-PP1.0-50-0.21	14.45	1.71	25.28	3.00
ACI-S0.5-50	32.80	3.90	20.81	2.47
ACI-S1.0-50	51.80	6.13	32.82	3.90
ACI-S0.5-38	34.30	4.00	24.70	2.92
ACI-S1.0-38	31.95	3.80	22.63	2.70
ACI-PP0.5-50	17.50	2.10	0.00	0.00
ACI-PP1.0-50	24.00	2.84	34.53	4.10

P_600_^d^: residual load at net deflection of L/600; F_600_^d^: residual stress at net deflection of L/600. P_150_^d^: residual load at net deflection of L/150; F_150_^d^: residual stress at net deflection of L/150.

**Table 9:** Published empirical relations between compressive strength and flexural strength.

ACI 318-14	Ahmed and Shah	Xu and Shi	Perumal
$ft=0.62{fc}^{0.5}$	$ft=0.44{fc}^{0.5}$	$ft=0.39{fc}^{0.59}$	$ft=0.259{fc}^{0.843}$

**Table 10:** Efficient indexes of the PPM mixtures.

PPM Mixtures	Efficient Indexes			Properties of FRC		
	IPS	MPT	FMF	VeBe Time (s)	Toughness (J)	Compressive Strength (MPa)
PPM-S0.5-50-0.26	0.59	1.00	0.03	7	63	36.4
PPM-S1.0-50-0.26	0.59	1.01	0.01	18	110	33.9
PPM-S0.5-50-0.21	0.45	1.03	0.02	12	80	33.9
PPM-S1.0-50-0.21	0.45	1.04	0.01	19	136	35.7
PPM-S0.5-38-0.26	0.59	1.00	0.04	6	42	33.2
PPM-S1.0-38-0.26	0.59	1.01	0.02	11	78	34.1
PPM-S0.5-38-0.21	0.45	1.03	0.03	11	80	32.6
PPM-S1.0-38-0.21	0.45	1.04	0.01	15	88	33.1
PPM-PP0.5-50-0.26	0.59	1.00	0.02	7	78	35.0
PPM-PP1.0-50-0.26	0.59	1.01	0.01	17	112	33.7
PPM-PP0.5-50-0.21	0.45	1.03	0.01	10	100	33.9
PPM-PP1.0-50-0.21	0.45	1.04	0.01	24	106	33.8

## Data Availability

The original contributions presented in this study are included in the article. Further inquiries can be directed to the corresponding author(s).

## Abbreviations

The following abbreviations are used in this manuscript:

ACI	American Concrete Institute
AV	Apparent viscosity
BC	Binder content
bi	Binder intensity factor
CA	Coarse aggregate
CPFT	Cumulative percentage finer than
CSA	Canadian Standards Association
df	Fiber diameter
Dmax	Maximum particle size
FA	Fine aggregate
FRC	Fiber-reinforced concrete
FF	Fiber factor
FMF	Fiber matrix factor
GU	General use
HB	Herschel–Bulkley
IPS	Interparticle separation distance
Lf	Fiber length
LF	Limestone filler
LCC	Low-cement concrete
MOR	Modulus of rupture
MPT	Maximum paste thickness
PC	Portland cement
PP	Polypropylene
PPM	Particle packing models
PSD	Particle size distribution
q-factor	Coefficient of distribution
RH	Relative humidity
SCMs	Supplementary cementitious materials
SP	Superplasticizer
VeBe	Vibrating table test time
Vf	Volumetric fiber fraction
VSA	Volumetric surface area
w/c	Water-to-cement ratio
XRF	X-ray fluorescence
